# Pleomorphic Adenoma of Base of Tongue: Is Midline Mandibulotomy Necessary for Approaching Benign Base Tongue Lesions?

**DOI:** 10.1155/2012/851501

**Published:** 2012-07-08

**Authors:** Sandeep Bansal, Gopika Kalsotra, Abdul Wadood Mohammed, Amanjit Bahl, Ashok K. Gupta

**Affiliations:** ^1^Departments of Otolaryngology and Head and Neck Surgery, Postgraduate Institute of Medical Education and Research, Chandigarh 160012, India; ^2^Departments of Histopathology, Postgraduate Institute of Medical Education and Research, Chandigarh 160012, India

## Abstract

*Objective*. To report a rare presentation of pleomorphic adenoma, at base tongue, excised surgically by a transoral midline glossotomy technique without mandibulotomy. *Case Report*. Pleomorphic adenoma is a benign tumor of the salivary gland found rarely in the base of tongue. Surgery is the definitive treatment for this tumor, and different approaches have been mentioned in the literature. In our case we surgically excised the tumor by a transoral midline glossotomy technique without mandibulotomy where we combined the cosmetic advantage of transoral technique and the exposure advantage of a glossotomy technique. *Discussion*. We discuss the different approaches to oropharynx, their advantages and disadvantages. Primary transoral approach provides better cosmesis but less exposure whereas median labiomandibuloglossotomy approach provides more exposure but is cosmetically unacceptable. *Conclusion*. A transoral midline glossotomy approach without mandibulotomy provides wide exposure with acceptable cosmesis.

## 1. Introduction

Pleomorphic adenoma or the benign mixed salivary gland tumor is the most common benign tumor of the major and minor salivary glands. It comprises about two-thirds of the salivary gland neoplasms. Even though it is predominantly found in the parotid gland, it forms the most common neoplasm in all salivary glands. The most common site of occurrence in the minor salivary glands is the palate. To date, only eight cases of pleomorphic adenoma involving the base of the tongue have been reported in the literature [1]. We report here our experience of a case of pleomorphic adenoma of the tongue base. We also discuss the incidence and diagnosis of base tongue pleomorphic adenoma and surgical approaches to this region.

## 2. Case Report

**Figure 1:**
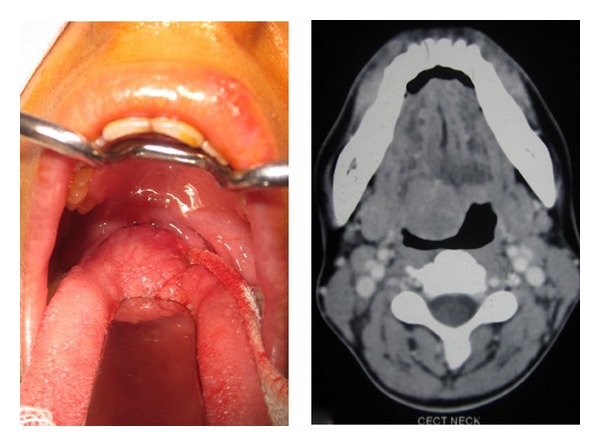
Figure showing the tumor in the base of tongue and the preoperative CT scan.

A 24-year-old female presented to our outpatient department with complaints of foreign body sensation of the throat for the last 1 year. She also complained of change in voice for the past 4 months. She had no associated history of dysphagia or difficulty in breathing. On examination there was a 3 × 3 cm smooth, grayish-white, firm, nontender swelling in the right side of base of tongue occupying the oropharynx (Figure 1). No cervical lymph nodes were palpable in the neck. Rest of examination of the ear, nose, and neck, as well as general physical examination, was unremarkable.

Contrast-enhanced computed tomography of the neck revealed a well-defined moderately enhancing soft tissue lesion involving the right side of base of tongue nearly 2.34 × 2.29 cm in size which was compromising the oropharyngeal airway. The thyroid gland was normally located in the anterior neck (Figure 1). Transoral fine needle aspiration cytology from the swelling was done which showed scattered cohesive clusters of epithelial cells surrounded by hyaline material. There was mild hyperchromasia and scattered squamous cells in background. Overall features suggested the possibility of pleomorphic adenoma.

**Figure 2:**
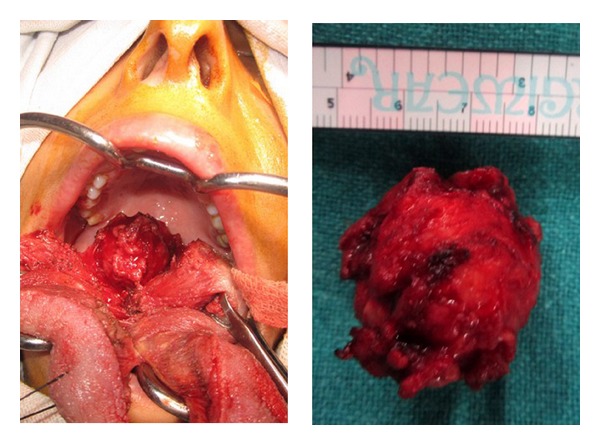
Intraoperative photograph showing midline glossotomy exposing the tumor in the base of tongue and removed specimen.

The patient was taken up for surgical excision. Tracheostomy was done prior to the procedure. A midline glossotomy was performed and deepened until the tumor was palpable. The lateral surface of the tumor was palpated and dissected using electrocautery. This approach provided an end on view to the lateral and inferior aspects of the tumor. The tumor was removed in toto with a cuff of normal tissue. The wound was sutured vertically in the midline from vallecula to tip of tongue (Figure 2). Patient was started on oral feeding on the first postoperative day and was decannulated on the 5th day.

**Figure 3:**
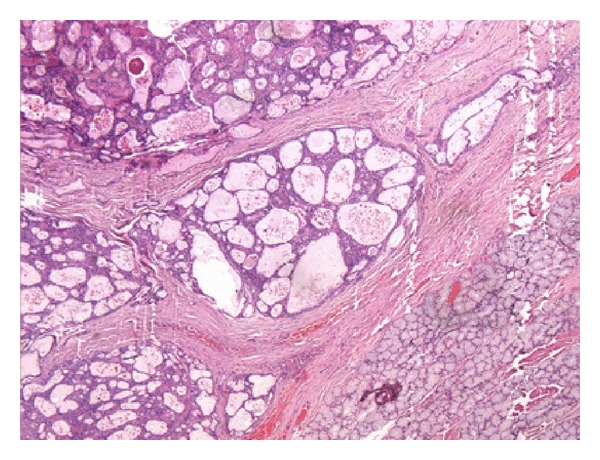
Microphotograph (H & E stain, ×200) showing epithelial and mesenchymal elements suggestive of pleomorphic adenoma.

Postoperative histopathology confirmed the diagnosis of pleomorphic adenoma with focal area of skeletal muscle involvement (Figure 3).

**Figure 4:**
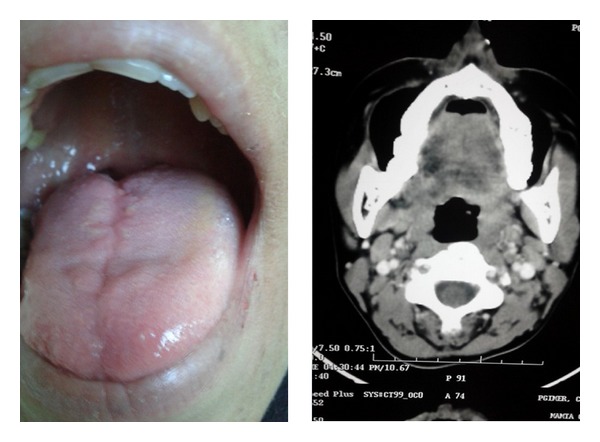
Appearance of tongue after 2 months following surgery and postoperative computed tomography.

A postoperative computed tomography was done after 2 months of the operation which showed complete removal of the tumor. There has been neither functional disturbance nor any signs of recurrence to date (Figure 4).

## 3. Discussion

Tumors of the salivary glands comprise 3% of all neoplasms [2]. The majority of salivary gland neoplasms are benign with pleomorphic adenomas being the most common. The incidence of neoplasms in minor salivary glands varies from 9 to 22% [3]. Approximately 8% of pleomorphic adenomas involve the minor salivary glands [2]. A study by Yoshihara and Suzuki found that the majority of pleomorphic adenomas involved the palate, followed by the lips [4]. Involvement of the base of the tongue was extremely rare. Malignant tumors involve the tongue more frequently than their benign counterparts. Only eight cases of pleomorphic adenoma involving the base of the tongue have been reported in the literature [1–8]. Most patients with pleomorphic adenoma of the base of the tongue either present with worsening dysphagia or it is detected on routine examination by general practitioners.

Treatment for pleomorphic adenoma is primarily surgical. Although these tumors are well encapsulated, resection of the tumor with an adequate margin is essential to avoid recurrence. Surgical approaches to the base of the tongue vary according to the size and site of the tumor and include transoral, combined transoral-transcervical, transpharyngeal, and transmandibular. Transpharyngeal can be either suprahyoid or transhyoid pharyngotomy or by lateral pharyngotomy. Transmandibular can be lip splitting, mandibular swing, or median labiomandibuloglossotomy.

Transoral approach is suited for small tumors which are exophytic. A tumor that extends too far inferiorly (i.e., past the tip of the epiglottis) or too far laterally cannot be accessed well with this approach. Difficulty in accessing the area and chance of bleeding and tumor spillage are other disadvantages of this approach. This approach can be augmented with laser or Da Vinci Robots. In the case report by Toshio Yoshihara [4], they have used transoral CO_2_ laser technique to resect a 2 × 3 cm exophytic pleomorphic adenoma. In another report by Grewal et al. [2], a pedunculated pleomorphic adenoma of 4 cm diameter and 3 cm stalk was removed by transoral approach. The patient had presented with respiratory distress.

The combined transoral-transcervical approach or the lingual-mandibular release can be used for base of tongue lesions. A floor of the mouth incision is made from one tonsillar pillar to the other. This releases the tongue and floor of the mouth in order to pull these structures below the mandible into the neck. The lingual arteries and nerve and the hypoglossal nerve are at risk in this approach. The suprahyoid pharyngotomy is where the entrance into the pharynx is made through the vallecula. This procedure allows preservation of the lingual arteries and nerve and the hypoglossal nerve. Extension of the pharyngotomy laterally and inferiorly along the thyroid ala allows wider exposure. Even though this technique provides excellent functional and cosmetic outcome, poor visualization of the superior margin of large tumors and in the worst cases the possibility of cutting into tumor is a major drawback. In the lateral pharyngotomy the pharynx is entered posterior to the thyroid ala. The hypoglossal and superior laryngeal nerves should be preserved carefully. This approach allows a good view of the posterior pharyngeal wall, opposite lateral wall, and base of tongue. If more superior exposure is needed, the pharyngotomy can be extended across the vallecula or this approach can be combined with a lateral mandibulotomy. But this would put the inferior alveolar nerve in jeopardy and risk of osteoradionecrosis if radiation is planned to this site. In a case report by Gupta et al. [5], a 2.5 × 1.75 cm pleomorphic adenoma of the base of tongue was excised through a lateral pharyngotomy approach. The patient was decannulated on the 10th day.

The mandibulotomy approach can be either a lip splitting or median labiomandibulotomy or a mandibular swing operation. The lip is split in the midline either vertically or by the modified zigzag-stepped technique which minimizes the risk of vermilion contracture. In the labiomandibulotomy approach the lip, gingiva, mandible, and anterior tongue are split in the midline. The incision can be carried through the base of tongue to the hyoid bone if exposure of the posterior pharyngeal wall is necessary. In the mandibular swing approach, the osteotomy is placed anterior to the mental nerve on the ipsilateral side at the site of a missing or extracted tooth. A cut is then made through the floor of the mouth until the anterior margin of resection is reached. The mandible and the tongue are then retracted exposing the tumor. The lingual nerve is usually sacrificed in this approach.

Our patient wanted a good cosmetic result being a young unmarried female. Taking that into consideration we decided to perform a transoral midline glossotomy without performing a median mandibulotomy. By this approach we could achieve the better cosmetic results of the transoral approach and the better exposure of a glossotomy. In the absence of the facility of a laser, this approach turned out to be very effective in our case. The tumor could be exposed in the lateral, inferior, and posterior planes and removed with adequate surgical margins. A tracheostomy was done anticipating postoperative tongue oedema, and patient was decannulated on the 5th day.

To conclude, oropharynx is a comparatively inaccessible region. Lesions of oropharynx are accessed by many approaches each having their own pros and cons. A transoral midline glossotomy approach without mandibulotomy provides wide exposure with acceptable cosmesis.

## 4. Summary

- Pleomorphic adenoma involving the base of the tongue is a rare entity.
- Base tongue is a comparatively inaccessible region, and many different approaches have been described each with their own advantages and disadvantages.
- A transoral midline glossotomy approach without mandibulotomy provides wide exposure with acceptable cosmesis.
